# Surgical Treatment of Upward Fixation of the Patella in a Multiparous Borana Breed Cow

**DOI:** 10.1155/2022/4929020

**Published:** 2022-04-19

**Authors:** Cheru Telila, Jiregna Dugassa

**Affiliations:** ^1^Department of Veterinary Teaching Hospital, College of Veterinary Medicine and Agriculture, Addis Ababa University, P.O. Box 34, Bishoftu, Oromia, Ethiopia; ^2^Department of Clinical Studies, College of Veterinary Medicine and Agriculture, Addis Ababa University, P.O. Box 34, Bishoftu, Oromia, Ethiopia

## Abstract

A 6-year-old multiparous Borana breed cow, which has been suffering from difficulty walking for 6 months, was presented to veterinary teaching hospital. As her previous history indicated, the lameness started during the second gestation, especially in the early morning, and subsides after a few hours of sunrise. She was completely cured after giving her second birth and a similar condition reappeared from the 5^th^ month of the third gestation until she gave birth. However, unexpectedly the disease aggravated after 2 days of parturition, and she was unable to walk and forced to stay indoors due to complete extension of the stifle joint. As a result, she was unable to lay down and maintained a permanent standing position for 2 months of aggravation. Clinical findings showed difficult mobilization of the right hind leg, which was locked in extension at the stifle joint, and the hoof was dragging on the floor. Based on history and clinical findings, upper fixation of the patella was diagnosed, and it was treated with median patellar desmotomy under local anesthetic infiltration. Finally, after the complete severing of the medial ligament and skin closure, the animal was able to walk normally, and the wound was healed uneventfully.

## 1. Introduction

Upper fixation of the patella is one of the main functional disorders of the stifle joint in cattle and horses. In young horses, this condition occurs mainly secondary to the beginning of training and is mainly related to their straight hind limb conformation [1]. It can be characterized by temporary or permanent dislocation of the patella from its regular position during locomotion. This problem is also commonly seen in young and debilitated animals with a steep angle between femur and tibia. In such animals, the condition is triggered by the tension brought by heavy work on the tendon and immature ligaments of the femuro-patellar articulation when they first start working as draft animals [2]. As stated by some authors, winter and summer seasons are aggravating factors for the upper fixation of the patella, though the disease is prevalent throughout the year [3, 4]. The disease is responsible for considerable economic loss as the lameness affects the working and feed searching ability of the bullocks and cows [2]. Upward fixation of the patella is treated surgically by medial patellar desmotomy. Diagnosis of this condition is mainly based upon anamnesis, clinical signs, and local palpation of the stifle joint [5, 6]. The current case report was aimed to describe successful surgical management of upward fixation of the patella in the cow that has been suffering for a long time and after formation of mild adhesion between the patella and trochlear ridge. Moreover, as it is the first report in Ethiopia, it is expected to increase the veterinary professional's consideration of the disease prevalence in the area.

## 2. Case Presentation and Treatment

**Figure 1:**
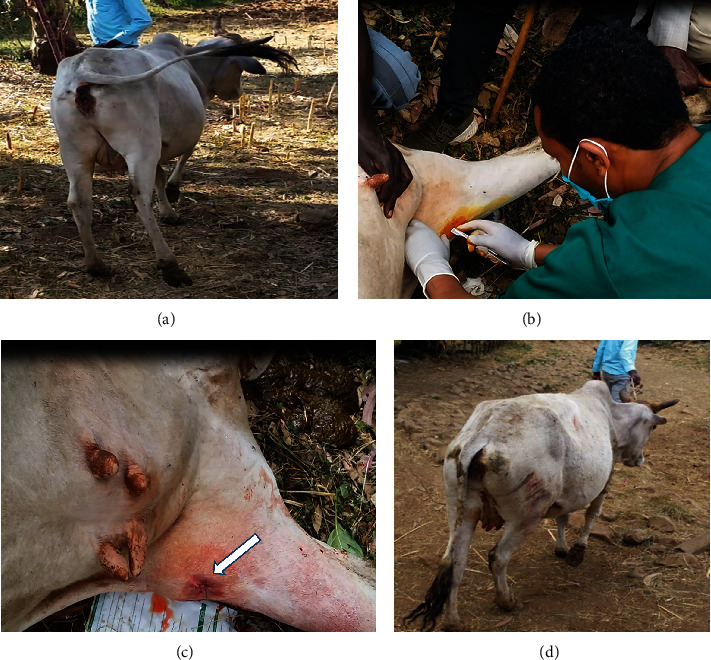
Clinical presentation, management of upper fixation of the patella, and its outcome. (a) Locked stifle joint and dragging right hoof. (b) Stab skin incision for medial patellar desmotomy. (c) After the closure of the skin incision. (d) Cow walking normally just after the procedure.

A 6-year-old Borana breed cow that has been suffering from difficulty walking for 6 months was presented for treatment. As her previous history indicated, the lameness was started during the second gestation. At that time lameness was observed daily in the early morning and subsides after a few hours of sunrise. She was completely cured after she gave birth to her second calf. But the condition reappeared similarly on the 5^th^ month of the third gestation until she calved. Contrary to the situation on the second parity, the condition was aggravated after 2 days of parturition. Consequently, she was unable to walk distances and forced to stay indoors rather than grazing in the field. The owner also expressed that she was unable to lay down and spent all the time in a standing position for the last 2 months of disease aggravation. After a long wait for her recovery, the owner called a nearby veterinary professional to visit her. Accordingly, she received antibiotics for two days suspecting blackleg and was finally referred to the Veterinary Teaching Hospital, Addis Ababa University. Clinical findings revealed good body condition, defensive behavior since she has calved, difficult mobilization of the right hind limb as it was locked in extension at stifle joint, and the tip of the hoof was dragging on the floor (Figure 1(a)). Additional physical examinations revealed that the stifle joint was not able to flex and remain rigid while the fetlock joint was able to flex normally. Finally, the case was diagnosed as an upward fixation of the patella, and the treatment was finalized to be through medial patellar desmotomy.

To perform desmotomy, the animal was controlled in lateral recumbency with the affected hind limb lowermost and extended caudally with a rope, while the two upper legs were tied together and held by assistants. The medial patellar ligament was located by upward sliding of the thumb on the cranial aspect of the tibia until the cranial tibial tuberosity was touched. The finger continued to move upward for about 3 cm until a thick middle ligament was palpated. Then, the finger was slightly slid toward the median site of the stifle joint into the depression located between the middle and medial patellar ligament. Finally, the broadest median ligament was identified by further sliding the finger to the medial border of the depression. This site was prepared for medial patellar desmotomy by hair shaving and routine surgical scrubbing. Then, 5 ml of 2% lidocaine was directly infiltrated at the surgical site.

It was decided to treat the case with an open technique of median patellar desmotomy. To perform this, a stab skin incision of about 2 cm long was made immediately at the lateral border of the medial ligament, 3 cm proximal to the tibial crest (Figure 1(b)). Following incision of skin and subcutaneous tissue, the left index finger was introduced to estimate the thickness of the ligament. Then, a number 20 scalpel blood fixed on the handle was introduced beneath the ligament, and the sharp end was held against the ligament to cut the whole thickness of the ligament toward the skin. While cutting the ligament, the surgeon kept the balance of his hand to avoid cutting to the overlying skin and other surrounding structures until crunching sound (sound heard when ligament cut) come to an end. The ligament was severed at its distal end to avoid damage to the stifle joint capsule.

Complete severing of the ligament was checked by external palpation of the depression formed at the previously tensed medial ligament or internally by checking if the two cut ends went apart, using the left index finger for the right leg. To prevent secondary infection, fortified procaine penicillin powder was added into the stab incision and closed with one simple interrupted suture (Figure 1(c)). Immediately after the patient was allowed to stand, she was able to walk normally by flexing her stifle joint (Figure 1(d)), with a slight crippling which gradually disappeared as she continued walking.

Postoperatively, the surgical site was disinfected with iodine tincture, after skin closure. The patient has additionally received diclofenac sodium at 1.5 mg/kg, IM, for two days. The owner was advised to refrain the animal from a long journey till full recovery. The suture was removed on the 8^th^ day of the surgical procedure, following complete healing of the surgical wound.

## 3. Discussion

Upward fixation of the patella is the most common clinical condition in the stifle joint in large animals [2, 5, 7]. Late pregnancy and early lactation are considered as predisposing factors in buffaloes [2], which is in agreement with the present finding, in which the cow was affected for the two consecutive gestation periods. It can be effectively treated by medial patellar desmotomy and can cure the affected animal completely if performed properly. Additionally, it provides immediate and permanent relief from the condition with minimum postoperative care [2]. This effective cure was currently attained by the work done in the Borana breed cow that has been suffering for 6 months and able to get immediate and permanent relief after the treatment.

As it was indicated by several studies, upward fixation of patella could be treated both by surgical (open) method and blind (closed) method [2, 5, 7–9]. However, some authors described as the open surgical management method requires more postoperative care and is time-consuming when compared to closed method [2, 4], but they did not indicate the length of time taken to complete the surgical procedure. Nevertheless, in the current case report, the time taken to conduct the procedure was only 6 minutes, starting from site preparation to skin closure but without including the time awaited for the local anesthetic to act, as it is common for both closed and open methods. Additionally, in this technique, special instruments were not used, except for the scalpel blade with handle, needle holder, and suture needle. The majority of the surgeons prefer the recumbent position for patellar desmotomy [10]. Similarly, the present procedure was conducted in the patient positioned in lateral recumbency to maximize the safety of the surgeon, as the animal was vicious even with the leg affected.

Even though it did not happen in the present case, the open method is suspected to have a higher risk of inducing surgical wound infection [4]. In the present technique, a small skin incision (2 cm) was made to sever the ligament, with great care without exteriorizing the ligament. But in other reports, a relatively large incision was made, and the ligament was severed after exteriorization [2, 10]. There was no literature describing the duration of upper fixation before surgical correction and the presence of adhesion between the patella and surrounding structures at the displaced position. However, the present surgical management was performed in a patient that has been suffering for a total of 190 days, while complete upward fixation (complete extension of stifle joint) lasted for 66 days. Consequently, after complete resection of the medial ligament, the resistance to flex the affected joint and rough gliding of the patella was appreciated during the first attempt of manual flexion. The roughness was reduced after 3 repeated manual flexion, and slight crippling was observed just at the time the animal started walking. The resistance was most probably has arisen from mild adhesion between the patella and medial trochlear ridge.

From this, it was concluded that if the animal remain untreated for a relatively long time after complete upper fixation, median patellar desmotomy could not be the sole treatment, because the firmness of patellar adhesion at the displaced position can increase with duration. Additionally, undergoing desmotomy through stab incision without exteriorization of the tensed median ligament was found an effective technique in terms of saving time, reducing wound size, and postoperative complications. Therefore, the authors recommend this promising surgical management technique for similar cases.

## Data Availability

The data used to support the findings of this study were included in the article, and additional data including videos can be provided upon request of the corresponding author.
